# Clinical and Imaging Features of Patients With Encephalitic Symptoms and Myelin Oligodendrocyte Glycoprotein Antibodies

**DOI:** 10.3389/fimmu.2021.722404

**Published:** 2021-10-07

**Authors:** Jingsi Wang, Zhandong Qiu, Dawei Li, Xixi Yang, Yan Ding, Lehong Gao, Aihua Liu, Yang Song, Cunjiang Li, Ran Gao, Lin Wang, Liyong Wu, Longfei Jia, Dongmei Guo, Aihong Zhou, Jianping Jia, Liyuan Huang, Miao Qu, Li Gao, Huiqing Dong, Junwei Hao, Zheng Liu

**Affiliations:** Department of Neurology, Xuanwu Hospital, Capital Medical University, Beijing, China

**Keywords:** myelin oligodendrocyte glycoprotein, encephalitis, autoimmunity, neuroimmunology, neuroimaging, leukodystrophy

## Abstract

**Background:**

Myelin oligodendrocyte glycoprotein-antibody (MOG-ab)-associated disease (MOGAD) has highly heterogenous clinical and imaging presentations, in which encephalitis is an important phenotype. In recent years, some atypical presentations in MOG-ab-associated encephalitis (MOG-E) have been increasingly reported but have not yet been described well. The aim of the study was to describe the clinical and imaging features of patients with MOG-E in our center. Atypical phenotypes would be reported, which is expected to expand the spectrum of MOGAD.

**Methods:**

We reviewed medical records of 59 patients with MOGAD diagnosed in our center and identified cases who had ever experienced encephalitic symptoms. Three hundred ten patients with autoimmune encephalitis (AE) were also reviewed, and cases with positive MOG-ab were identified. Besides, patients with chronically progressive encephalitis were identified from 13 MOG-E and 310 AE patients. We collected demographic, clinical, laboratory, radiological, and outcome data to explore clinical and imaging characteristics in MOG-E, especially in the atypical phenotype of chronically progressive encephalitis.

**Results:**

We identified 13 patients (7 males, 6 females) with MOG-E. The median age at onset was 33 years (range 13~62 years). Most (9/13, 69.2%) of patients showed acute or subacute onset of encephalitic symptoms. Brain MRI abnormalities were observed in all patients. The most common lesion locations on MRI were cortical/subcortical (11/13, 84.6%), deep/periventricular white matter (10/13, 76.9%) and corpus callosum (4/13, 30.8%). Brain MRI patterns were categorized into four phenotypes. The most common pattern was cortical encephalitis with leptomeningeal enhancement/brain atrophy (10/13, 76.9%). Eight (8/13, 61.5%) patients had a good response to immunotherapy. Four (4/13, 30.8%) patients with chronically progressive course were identified from MOG-E cohort. They showed leukodystrophy-like pattern, multifocal hazy lesions, or cortical encephalitis on MRI. With immunotherapy, they only showed mild or no improvement. We also identified four (4/310, 1.3%) patients with chronically progressive course from AE cohort. They had better outcomes than counterparts in MOG-E.

**Conclusions:**

This study demonstrates that encephalitic presentations in MOGAD had complex clinical patterns. Chronically progressive encephalitis may be a new phenotype of MOGAD. We recommend to test MOG-ab in subacute and chronic progressive dementia with leukodystrophy-like MRI lesions.

## Introduction

Myelin oligodendrocyte glycoprotein-antibody (MOG-ab)-associated disease (MOGAD) or MOG-immunoglobulin G (MOG-IgG)-associated encephalomyelitis (MOG-EM) has been recognized as a distinct neuroimmunological disorder with specific clinical features and management (1). With the wide availability of cell-based assays (CBAs), MOG-ab was described in a variety of demyelinating syndromes (1). According to previous studies, acute disseminated encephalomyelitis (ADEM)-like presentation is more common in children and opticospinal presentation is more common in adults (2).

When brain is affected by MOG-ab, the encephalitic symptoms can be similar to ADEM, especially in children (3–6). In the past few years, patients with MOG-ab and encephalitis or encephalitic symptoms who did not fulfill the criteria of ADEM were reported in several research (4, 7, 8). Characteristics of encephalitic symptoms (such as seizures, psychiatric symptoms, and cognitive impairment) were described in MOGAD in recent studies (9–14). Most of the patients had acute or subacute onset, but slowly progressive course was also reported (13, 15). In imaging studies, cortical gray matter, subcortical white matter, periventricular white matter, and deep gray matter were frequently involved (11, 16, 17), and “leukodystrophy-like” patterns were also reported (14). In prognosis, most patients showed a good response to immunotherapy (2), but highly relapsing and treatment-resistant patients were reported as well (18, 19).

The spectrum of MOGAD is expanding. The encephalitic syndrome is recognized as an important component, which was found more common in MOGAD than in neuromyelitis optica spectrum disorders (NMOSD) (9, 11, 17). Herein, we report 13 cases with encephalitic symptoms and positive MOG-ab to describe the clinical and imaging features of MOG-ab-associated encephalitis (MOG-E). Of note, atypical presentations, chronically progressive encephalitic symptoms with leukodystrophy-like pattern and brain atrophy on MRI, were also described in this study, which are expected to expand clinical and imaging phenotypes in MOGAD and to prompt the testing of MOG-ab in patients with chronically progressive encephalitic symptoms and leukodystrophy-like MRI pattern.

## Methods

### Patients

We retrospectively reviewed the medical records of 59 patients from Xuanwu Hospital, Beijing, China, between March 2017 and August 2021 who fulfilled the criteria of MOG-EM or possible MOG-EM (1). According to the criteria, all of these patients showed clinical or paraclinical evidence of central nervous system (CNS) demyelination and were tested positive for MOG-ab. Patients who had ever experienced encephalitic symptoms (cognitive impairment, seizures, psychiatric symptoms, and/or decreased level of consciousness) were identified and followed up.

Simultaneously, 310 patients who fulfilled the criteria for autoimmune encephalitis (AE) (20) during the same period were also reviewed. All of the patients experienced encephalitic attacks. Cases with positive MOG-ab were identified and followed up.

**Figure 1:**
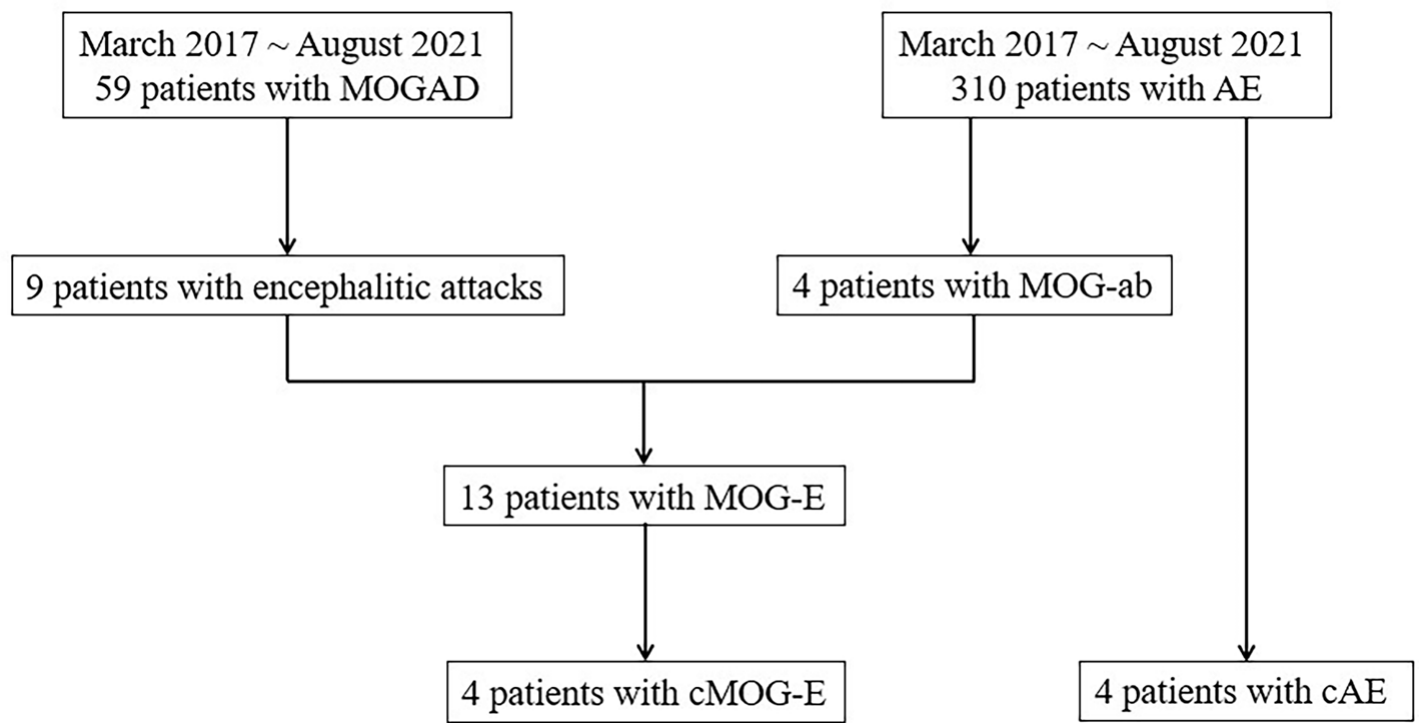
The process of identifying patients from MOGAD and AE cohorts. ab, antibodies; AE, autoimmune encephalitis; cAE, chronically progressive autoimmune encephalitis; cMOG-E, chronically progressive myelin oligodendrocyte glycoprotein-antibodies-mediated encephalitis; MOG, myelin oligodendrocyte glycoprotein; MOGAD, myelin oligodendrocyte glycoprotein-antibodies-associated disease.

Four patients with chronically progressive MOG-E (cMOG-E) were identified and focused on, which was defined as encephalitic symptoms started insidiously and progressed slowly (more than three months). Chronically progressive AE (cAE) were identified from 310 AE patients to make a comparison in clinical and imaging characteristics. **Figure 1** demonstrates the process of identifying patients in the study.

Demographic characteristics, the form of disease onset and progression, disease duration at presentation, clinical presentations, laboratory results, imaging findings, immunotherapy, period of follow-up, disease course and evolution, outcomes at the last follow-up were described. Disease severity at nadir of encephalitic attacks and neurological outcomes at the last follow-up were assessed with the modified Rankin Scale (mRS), Expanded Disability Status Scale (EDSS), and Mini Mental State Examination (MMSE). This study was approved by the ethics committee of the Xuanwu hospital, Capital Medical University. All patients provided written informed consent.

### Laboratory Studies

Lumbar puncture was performed at the earliest available time in all patients. Cerebrospinal fluid (CSF) white cell count, protein concentration, and intrathecally restricted oligoclonal bands (OB) were recorded. In all patients, MOG-ab, aquaporin 4-ab (AQP4-ab), and antibodies associated with autoimmune encephalitis (AE) and paraneoplastic neurological syndromes (PNS) were assessed by live CBAs analyzed by flow cytometry in serum and CSF as routine workup for suspected neuroimmune disorders and encephalitis. Serum antinuclear antibodies (ANA), antineutrophil cytoplasmic antibodies, anticardiolipin antibodies, and rheumatic factor (RA) were tested in all patients to exclude systemic autoimmunity and to seek concomitant autoantibodies. CSF from all patients were tested for viruses and bacterium to exclude infections in CNS.

### Imaging

Brain magnetic resonance imaging (MRI) was performed in all patients, including T1-weighted, T2-weighted, fluid-attenuated inversion recovery (FLAIR), and diffusion-weighted imaging. Gadolinium-enhanced T1-weighted imaging was available in 12 patients. Brain positron emission tomography (PET) was performed in two patients.

Abnormalities on brain MRI and PET were described regarding locations, such as cortical gray matter, subcortical white matter, periventricular white matter, corpus callosum, brainstem, brachium pontis, thalamus, cerebellum, and hippocampus. Gadolinium enhancement of lesions was also assessed.

Hacohen et al. (14) classified MRI lesions in pediatric MOGAD into four types. Combined with MRI characteristics in our patients, we categorized abnormalities on brain MRI in our MOG-E patients into four patterns: (I) multifocal hazy/poorly marginated lesions, involving both gray matter and white matter and typically involving the middle cerebellar peduncles; (II) extensive and periventricular white matter lesions resembling a “leukodystrophy-like” pattern; (III) cortical encephalitis with leptomeningeal enhancement/brain atrophy; (IV) tumefactive demyelinating lesions (TDLs).

## Results

All of 59 patients who were diagnosed as MOGAD in the Department of Neurology of Xuanwu Hospital experienced clinical attacks suggesting CNS demyelination. MOG-ab were detected by live CBAs in all 59 patients, and the titer was between 1:100 and 1:320 (median 1:100). Nine patients (9/59, 15.3%) who had ever experienced encephalitic symptoms during attacks were included in this study.

In 310 patients who were diagnosed as AE, four patients were tested positive for MOG-ab and were included in this study. Three out of them had coexistent anti-N-methyl-D-aspartate receptor (NMDAR)-ab. In total, we identified 13 cases with MOG-ab who had ever experienced encephalitic symptoms during their disease courses, among whom three patients had more than one episode of encephalitic attack (**Tables S1** and **S2**).

**Figure 2:**
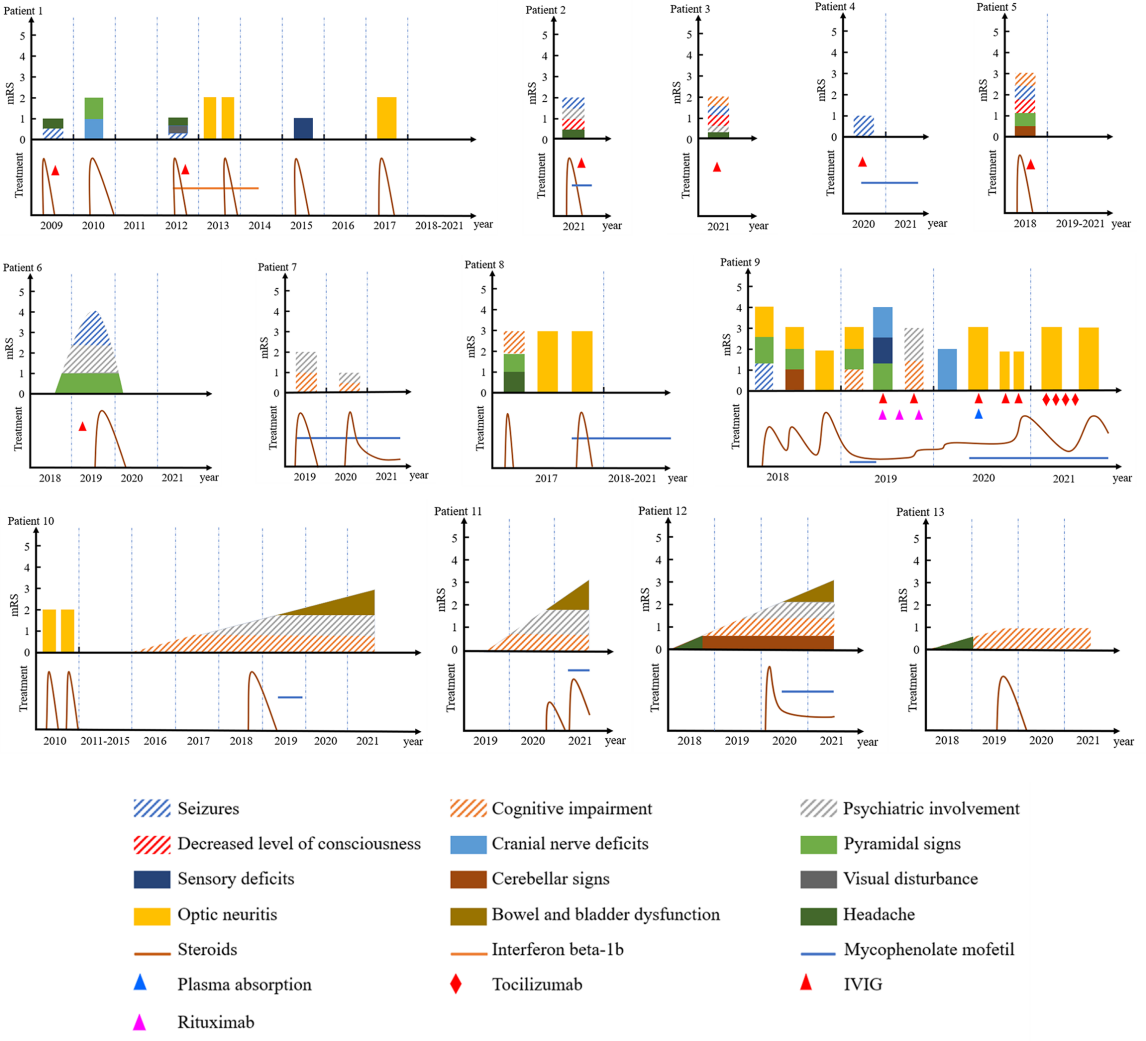
Clinical courses and treatment in each MOG-E patient. Bars of encephalitic symptoms (seizures, cognitive impairment, psychiatric symptoms, and/or decreased level of consciousness) are shadowed. IVIG, intravenous immunoglobulin; mRS, modified Rankin scale.

The demographic, clinical, treatment, and follow-up data of the 13 patients are summarized in **Table 1**. **Figure 2** demonstrates clinical courses and treatment in every patient. Two patients were <18 years at disease onset. Most of patients had an acute or subacute onset with rapid progression of encephalitic symptoms, but four patients (patients 10, 11, 12, and 13) showed an insidious onset with slow progression over 3 months. All patients received immunotherapy, and immunosuppressant was given to nine patients. Most patients had a good response to treatment, but four patients only showed mild response (patients 10, 11, and 13) or even no response (patient 12).

**Table 1:** Demographic, clinical, treatment and follow-up data in 13 patients with MOG-E.

Characteristic	Value
Number of patients, n	13
Male/Female, n	7:6
Age at onset, years, median (range)	33 (13~62)
<18 years at onset, n	2
Disease duration at presentation, months, median (range)	12 (0.2~89)
Preceding infection, n/total (%)	4/13 (30.8%)
Form of onset	
Acute, n/total (%)	7/13 (53.8%)
Subacute, n/total (%)	2/13 (15.4%)
Insidious, n/total (%)	4/13 (30.8%)
Form of symptom progression	
Rapid, n/total (%)	8/13 (61.5%)
Chronic, n/total (%)	5/13 (38.5%)
Symptoms	
Decreased level of consciousness, n/total (%)	3/13 (23.1%)
Cognitive dysfunction, n/total (%)	9/13 (69.2%)
Psychiatric symptoms, n/total (%)	8/13 (61.5%)
Seizure, n/total (%)	7/13 (53.8%)
Other neurological symptoms, n/total (%)	11/13 (84.6%)
Clinical assessment score at nadir of attack	
mRS, median (range)	3 (1~4)
EDSS, median (range)	2.5 (0~8.5)
MMSE, median (range)	24 (3~29)
Immunotherapy	
IVMP and/or IVIG, n/total (%)	13/13 (100%)
Immunosuppressant, n/total (%)	9/13 (69.2%)
MMF, n	8
Interferon beta-1b*, n	1
Rituximab, n	1
Follow-up, months, median (range)	24 (3~53)
Clinical assessment score at last follow-up	
mRS, median (range)	0 (0~3)
EDSS, median (range)	0 (0~6)
MMSE, median (range)	28 (3~29)
Response to immunotherapy	
Good response, n/total (%)	8/13 (61.5%)
Partial response, n/total (%)	1/13 (7.7%)
Mild recovery, n/total (%)	3/13 (23.1%)
No response, n/total (%)	1/13 (7.7%)
Disease course	
Monophasic, n/total (%)	6/13 (46.2%)
Relapsing, n/total (%)	4/13 (30.8%)
Highly relapsing, n	2
Progressive, n/total (%)	3/13 (23.1%)

EDSS, Expanded Disability Status Scale; MMF, mycophenolate mofetil; MMSE, Mini Mental State Examination; MOG-E, myelin oligodendrocyte glycoprotein-ab-associated encephalitis; mRS, modified Rankin scale; n, number of cases; IVIG, intravenous immunoglobulin; IVMP, intravenous methylprednisolone.

*Interferon beta-1b was used in patient 1 for a misdiagnosis of multiple sclerosis.

Laboratory and radiological findings of the 13 patients are shown in **Table 2**. Three patients (patients 4, 5, and 7) had overlapping MOG-ab and NMDAR-ab. Two patients (patients 6 and 7) were tested positive for other autoantibodies. Concomitant systemic autoimmunity was reasonably excluded in all patients.

**Table 2:** Laboratory and radiological findings in 13 patients with MOG-E.

Characteristic	Value
CSF	
Pleocytosis, n/total (%, median, range)	8/13 (61.5%, 25.5, 9~180)
Elevated protein level, n/total (%, median, range)	5/13 (38.5%, 52, 47~62)
SOB, n/total (%)	3/13 (23.1%)
MOG-IgG	
Positive in serum, n/total (%)	13/13 (100%)
Titer in Serum, median (range)	1:100 (1:100~1:320)
Positive in CSF, n/total (%)	9/13 (69.2%)
Titer in CSF, median (range)	1:10 (1:1~1:100)
NMDAR-IgG	
Positive in serum, n/total (%)	1/13 (7.7%)
Positive in CSF, n/total (%)	3/13 (23.1%)
Other positive antibodies in serum	
ANA, n/total (%)	1/13 (7.7%)
Anti-β2-GPI, n/total (%)	1/13 (7.7%)
Anti-amphiphysin antibodies, n/total (%)	1/13 (7.7%)
RF, n/total (%)	1/13 (7.7%)
Brain MRI lesions	
Presence of brain lesion, n/total (%)	12/13 (92.3%)
Cortical/subcortical, n/total (%)	11/12 (91.7%)
Deep white matter, n/total (%)	5/12 (41.7%)
Periventricular white matter, n/total (%)	5/12 (41.7%)
Corpus callosum, n/total (%)	4/12 (33.3%)
Brainstem, n/total (%)	3/12 (25.0%)
Brachium pontis, n/total (%)	1/12 (8.3%)
Thalamus, n/total (%)	1/12 (8.3%)
Cerebellum, n/total (%)	1/12 (8.3%)
Brain atrophy on MRI	
Presence of brain atrophy, n/total (%)	4/13 (30.8%)
Whole-brain atrophy, n	3
Bilateral hippocampi atrophy, n	1
Gadolinium enhancement on MRI, n/total (%)	4/12 (33.3%)
Brain MRI patterns	
I. Multifocal hazy/poorly marginated lesions	5/13 (38.5%)
II. Leukodystrophy-like pattern	2/13 (15.4%)
III. Cortical encephalitis with leptomeningeal enhancement/brain atrophy	10/13 (76.9%)
IV. Tumefactive demyelinating lesions	2/13 (15.4%)
Lesions in the spinal cord, n	0

β2-GPI, β2-glycoprotein I; ANA, antinuclear antibody; CSF, cerebrospinal fluid; IgG, immunoglobulin; MRI, magnetic resonance imaging; MOG, myelin oligodendrocyte glycoprotein; n, number of cases; MOG-E, myelin oligodendrocyte glycoprotein-ab-associated encephalitis; NMDAR, N-methyl-D-aspartate receptor; RF, rheumatoid factor; SOB, specific oligoclonal bands.

**Figure 3:**
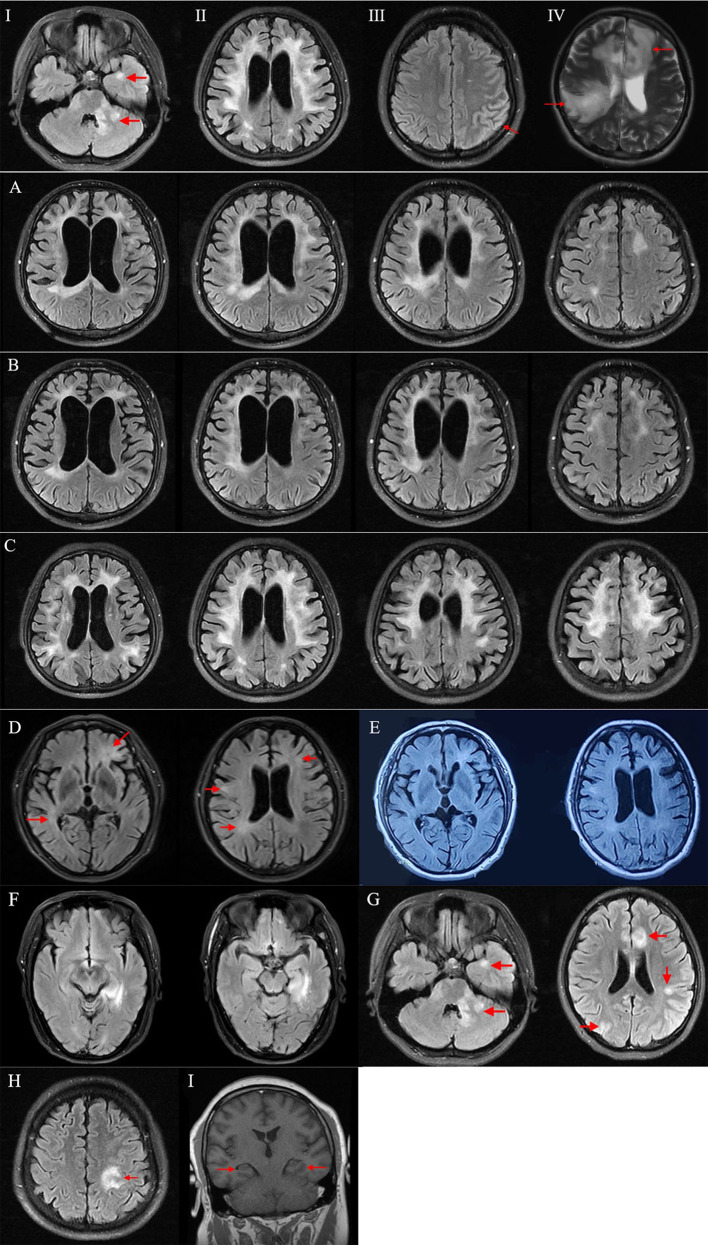
Brain MRI in MOG-E patients. **(I–IV)**: Representative images of four brain MRI patterns in MOG-E. **(I)** Multifocal hazy/poorly marginated lesions; **(II)** leukodystrophy-like pattern; **(III)** cortical encephalitis with leptomeningeal enhancement/brain atrophy; **(IV)** tumefactive demyelinating lesion. **(A–F)**: Brain MRI in chronically progressive MOG-E. **(A)** Brain MRI of patient 10 in 2018 showed leukodystrophy-like white matter change with whole-brain atrophy. **(B)** Brain MRI of patient 10 in 2019 showed slight improvement of white matter change on MRI. **(C)** Brain MRI of patient 11 showed leukodystrophy-like pattern with whole-brain atrophy. **(D)** Brain MRI of patient 12 in 2020 showed multifocal hazy and poorly marginated lesions. **(E)** Brain MRI of patient 12 in 2021 showed no prominent improvement of lesions. **(F)** Brain MRI of patient 13 showed a lesion in the left hippocampus and parahippocampal gyrus. **(G–I)**: Brain MRI abnormalities in patients with overlapping MOG-ab and NMDAR-ab. **(G)** Patient 5 showed multifocal poorly marginated lesions in cortical gray matter, subcortical white matter, and midline structures on MRI. **(H)** Patient 4 showed cortical and subcortical lesions in the left frontal and parietal lobes on MRI. **(I)** Patient 7 showed mild bilateral hippocampi atrophy without lesion on MRI. ab, antibodies; MOG-E, MOG-antibody-associated encephalitis.

In imaging, all patients showed abnormalities (**Supplementary Figures 1, 2**). Brain lesions were seen on MRI in 12 patients. In one patient (patient 7), only bilateral hippocampi atrophy was revealed on MRI. Cortical/subcortical, deep/periventricular white matter, and corpus callosum were the most common lesion locations in this cohort. MRI abnormalities in every patient were classified as one or two patterns, and representative images in every MRI pattern were shown in **Figures 3I–IV**. The most common brain MRI pattern was cortical encephalitis with leptomeningeal enhancement/brain atrophy. Leukodystrophy-like and tumefactive demyelinating lesions were less common, but were also observed in this study.

Patients 10 and 13 had brain PET and showed hypometabolism in lesion and atrophy locations. Cerebellum hypometabolism was also observed in the two patients without cerebellar lesions or atrophy on MRI. Patient 7 had symptoms of cerebellar ataxia. Detailed information of the 13 MOG-E patients are provided in **Supplementary Tables 1, 2**.

Four (patients 10, 11, 12, and 13) out of 13 patients (30.8%) had insidious onset and chronically progressive disease course, which is atypical and rare in MOGAD. We present case summaries of the four patients as follows. Alternative diagnosis was excluded reasonably in all patients.

### Patient 10

A 32-year-old male presented with slowly progressive mood changes, abnormal behaviors, and short-term memory decline for 2 years. He had a history of two episodes of optic neuritis (ON) at the age of 24 with complete recovery after being treated with intravenous methylprednisolone (IVMP) and oral prednisolone tapering for 2 weeks. At presentation, his brain MRI showed confluent bilateral white matter abnormalities, remarkable whole-brain atrophy, enlarged ventricles, and hydrocephalus (**Figure 3A**). His MMSE was 28 and mRS was 2. CSF study showed 5 cells/ul, protein 35 mg/dl, and negative OB. MOG-ab were detected in his serum (titer 1:100), and he was treated with 1 g of intravenous methylprednisolone for 5 days followed by 80 mg of oral prednisolone taper for 6 months. Although his symptoms deteriorated gradually, his brain MRI showed slight improvement of white matter change (**Figure 3B**). Then he started on mycophenolate mofetil (MMF) but stopped it in the 12^th^ month of follow-up because of a bad response. At the follow-up by telephone in the 34^th^ month, his symptoms deteriorated prominently. His MMSE dropped to 3, and mRS was 3.

### Patient 11

A 57-year-old female presented with memory decline and personality and behavior change for 2 years. Her symptoms started insidiously and progressed chronically. Her brain MRI showed leukodystrophy-like white matter abnormalities with whole-brain atrophy (**Figure 3C**). CSF showed 1 cell/ul, protein 51.2 mg/dl, and negative OB. At presentation, her MMSE was 5 and mRS was 3. MOG-ab was positive in her serum (titer 1:100) and CSF (titer 1:10). She was treated with two cycles of high-dose IVMP following oral prednisolone tapering and MMF. At the follow-up in the 4^th^ month, the patient showed mild improvement of psychiatric symptoms. Her MMSE and mRS remained unchanged.

### Patient 12

A 54-year-old male presented with personality change, memory decline, and gait imbalance for 2 years. His brain MRI showed multifocal hazy and poorly marginated lesions with mild whole-brain atrophy (**Figure 3D**). CSF analysis revealed 31 cells/ul, protein concentration 62 mg/dl, and negative OB. His MMSE was 15 and mRS was 3. Testing for MOG-ab was positive in his serum (titer 1:320). He was treated with 1 g of IVMP following 60 mg of oral prednisolone tapering and MMF. At the follow-up in the 14^th^ month, his symptoms worsened, and no improvement was acquired on MRI (**Figure 3E**). His MMSE was 10 and mRS was 3.

### Patient 13

A 60-year-old female insidiously presented with memory decline for 1 year. Her symptoms showed a slow progression. On MRI, she had a lesion in the left hippocampus and parahippocampal gyrus (**Figure 3F**). CSF tests revealed 2 cells/ul, protein 23 mg/dl, and negative OB. Her MMSE was 24 and mRS was 1. Treated with 500 mg of IVIG for 5 days followed by oral prednisolone-tapering for 3 months, her symptoms stabilized without further deterioration or improvement. Her MMSE and mRS remained stable.

In 310 AE patients, we identified four patients with chronically progressive AE (cAE) (**Table 3**). There were two males and two females. Three of them had positive leucin-rich glioma-inactivated 1 protein (LGI1)-ab, and one patient had positive glutamate decarboxylase 65 (GAD65)-ab. Three out of the four patients developed dystonia or seizures after months of slowly progressive cognitive impairment and/or psychiatric symptoms. They were all treated with high-dose IVMP followed by oral prednisolone tapering, and encephalitic symptoms in all patients were improved remarkably. On MRI, three patients showed normal results, and one patient showed a lesion in the right parietal lobe. CSF tests were normal in all patients. The median length of follow-up was 13 months (range 11~23 months). None of four patients relapsed during follow-up period.

**Table 3:** Demographic, clinical, imaging, characteristics, and treatment of four patients with cAE.

Patient	cAE-1	cAE-2	cAE-3	cAE-4
Positive AE antibodies	LGI1-ab 1:32 in serum	LGI1-ab 1:100 in serum	GAD65-ab 1:100 in serum and CSF	LGI1-ab 1:100 in CSF
Age (y)/Sex	76/M	58/F	39/F	44/M
Disease duration (months)	12	33	4	7
Chief complaint	Memory decline for 1 year and body twitching for 1 month.	Mood lability, personality change, and memory decline for 33 months, and paroxysmal hand tremor for 9 months.	Memory decline for 4 months and being sleepy for 2 weeks.	Memory decline and mood lability for 7 months and paroxysmal unconsciousness and limb twitching for 50 days.
mRS/EDSS/MMSE at nadir	1/2/25	3/3/19	1/2/25	1/2/26
Immunotherapy	Steroids	Steroids	Steroids	Steroids
CSF results				
White blood cells (/ul)	2	1	5	5
Protein (mg/dl)	45	19	25	26
Positive SOB	Neg	Neg	Neg	Neg
MRI features	Normal	Normal	A lesion in the right parietal lobe	Normal
Follow-up (months)	23	15	11	11
mRS/EDSS/MMSE at last follow-up	1/1/28	1/2/21	1/1/28	1/1/29
Outcome at last follow-up	Full recovery	Remarkable improvement	Remarkable improvement	Remarkable improvement
Disease course	Monophasic	Monophasic	Monophasic	Monophasic

ab, antibodies; AE, autoimmune encephalitis; cAE, chronically progressive autoimmune encephalitis; EDSS, Expanded Disability Status Scale; F, female; GAD65, glutamate decarboxylase 65; LGI1, leucin-rich glioma-inactivated 1 protein; M, male; MMSE, Mini Mental State Examination; mRS, modified Rankin scale; n, number of cases; SOB, specific oligoclonal bands; y, year.

## Discussion

MOG, produced by oligodendrocytes, is a protein that is specifically expressed on the outer surface of the myelin sheath in the CNS (2, 21). Although the biological function of MOG has not been explained thoroughly, it has already been used to induce a variety of experimental models, such as experimental autoimmune encephalomyelitis (EAE), which confirms its encephalitogenic property (2). Oligodendrocytes are not only present in the subcortical white matter but also exist in cortical gray matter, where they function in axonal conduction (21). This may explain the cortical symptoms in MOGAD.

Clinical and imaging features of the 13 patients with MOG-ab and encephalitic symptoms are described in our study. Two children with MOG-E had similar features with adult patients, which indicates that although ADEM is the main phenotype in pediatric MOGAD (2), encephalitis is also an important component. In the study of Armangue et al. (4), MOG-ab were the most common (13%) autoantibodies in pediatric AE, surpassing all neuronal antibodies combined. Therefore, testing of MOG-ab is necessary in pediatric encephalitis.

Generally, more than one-third of MOG-E patients in our cohort had classic encephalitic presentations, which are in accordance with previous descriptions (4, 7, 8, 17, 22). Most of them exhibited acute or subacute encephalitic symptoms with a good outcome after immunotherapy. On MRI, cortical/subcortical, deep/periventricular white matter, and corpus callosum lesions were frequently involved. Cortical encephalitis with leptomeningeal enhancement/brain atrophy was the most common MRI pattern in this cohort. However, atypical clinical and radiological characteristics were observed as well, which would be discussed below.

Four out of 13 patients in our study showed insidious onset and chronic progression of encephalitic symptoms (cognitive impairment and/or psychiatric symptoms). On MRI, leukodystrophy-like pattern with whole-brain atrophy, multifocal hazy/poorly marginated lesions, and cortical encephalitis were observed (**Figures 3A–F**). With the treatment of steroids and/or MMF, encephalitic symptoms and/or MRI abnormalities in three patients (patients 10, 11, and 13) were slightly improved. But symptoms in patient 12 deteriorated, and MRI lesions did not change at the last follow-up.

According to previous studies, chronic progression was very rare in MOGAD (1). Jarius et al. detected serum MOG-ab in none of 200 patients with primary/secondary progressive MS (23). We found seven cases with MOG-ab and chronically progressive disease course in previous English literature (**Table 4**) (13, 15, 18, 24–26). Five out of the seven patients had cognitive and psychiatric symptoms as the main progressive symptoms, and four patients showed poor response to immune treatment. Combined with our four patients, it is possible that chronically progressive course is a risk factor of bad prognosis. MOGAD is a distinct disease entity, but it shares some similarities in pathology and pathogenesis with MS in which neurodegeneration is independent of inflammatory relapses and inflammation interacts with neurodegeneration during the whole disease course (2, 18, 27–30). Progression may present when the loss of axons surpasses the compensatory capacity of the brain (29–32). It is possible that these mechanisms can partly explain chronic progression and irreversible disability in patients 10, 11, and 12. Besides, patient 13 and two patients in previous literature with MOG-ab and slowly progressive symptoms had a good outcome with immunotherapy, which might be attributed to earlier diagnosis and treatment (24, 25).

**Table 4:** Clinical and imaging characteristics of seven patients with positive MOG-ab and chronically progressive disease course in previous literature.

Literature	Age/sex	Progressive symptoms	Disease duration	Brain MRI features	Immunotherapy	Treatment response
Gil-Perotin et al.	32/M	Slowly progressive ataxia, brainstem symptoms, and urinary control after two attacks of ON	10 years	Cortical, juxtacortical, and multiple periventricular lesions at the level of lateral ventricles with predominant infratentorial lesions; cortical brain atrophy.	MP, IFN, GA, MX, RTX	Poor response
Yılmaz et al.	10/F	Gradually increased behavioral and personality changes after a focal onset seizure.	2.5 months	ADEM-like lesions	IVIG	Good response; complete clinical recovery
Baba et al.	60/M	Progressive cognitive deterioration and behavioral changes.	9 months	ADEM-like lesions	IVMP followed by oral prednisolone	Poor response: no improvement on symptoms; slight improvement on MRI.
Kwon et al.	43/M	Progressive dysarthria, dysphagia and gait disturbance after seven repeated ON attacks.	5 years	Progressive brain atrophy and white matter changes	Steroids, MMF, and IVIG.	Poor response
Papathanasiou et al.	57/F	Progressive cognitive decline and behavioral changes followed by a generalized seizure.	3 months	Wide pachymeningeal enhancement and hyperintense signal in both hippocampi.	Steroids	Good response: complete recovery on symptoms and MRI
Yazbeck et al.	15/M	Progressive cognitive degradation, behavioral difficulties, and seizures after postinfectious cerebellitis and ON.	8 years	Extensive leukodystrophy like lesions with gadolinium enhancement	Natalizumab, IVMP, RTX, and IVIG	Partial response: cognitive stabilization with slight improvement
	9/M	Progressive behavioral and cognitive impairment, epilepsy, and left hemiparesis	4~5 years	Leukodystrophy-like lesions	IVMP, IFN-b1a, mitoxantrone, RTX, and IVIG	Poor response: no improvement was observed

ADEM, acute disseminated encephalomyelitis; F, female; GA, glatiramer acetate; IFN, interferon; IVIG, intravenous immunoglobulin; IVMP, intravenous methylprednisolone; M, male; MMF, mycophenolate mofetil; MP, methylprednisolone; MX, mitoxantrone; ON, optic neuritis; RTX, rituximab.

In imaging, MRI patterns I, II, and III were observed in patients with chronically progressive course. Notably, two of them showed leukodystrophy-like lesions and whole-brain atrophy. In 2018, Hacohen et al. reported leukodystrophy-like MRI pattern in seven pediatric MOGAD who were younger than 7 years of age (14). Since then, similar cases were reported, but mostly in children. In our study, we observed leukodystrophy-like MRI pattern in two adults with MOG-ab. We identified some differences between adult and pediatric patients. Pediatric MOGAD patients with leukodystrophy-like MRI pattern in the study of Hacohen et al. had acute or subacute onset with rapid progression of symptoms. Although four patients relapsed with disease-modifying treatment, they can still get clinical improvement after treatment of steroids in acute phase. On MRI, gadolinium-enhancement was observed in leukodystrophy-like lesions. However, the two patients in our study showed chronically progressive course without acute attacks. With high-dose steroids pulses followed by oral prednisolone taper, they only got slight or even no improvement. On MRI, gadolinium-enhancement was absent in both two patients. These demonstrated that adult and pediatric MOG-E patients with leukodystrophy-like MRI pattern have different clinical, radiological, and prognostic characteristics. Generally, outcomes in adult and pediatric patients were poor, but adult appeared to have a worse outcome than children.

In the four patients with cMOG-E, patients with different MRI features had different outcomes. Patient 13 showed better outcome than patients 10, 11, and 12. One reason might be that the case only had one focal lesion without brain atrophy on MRI, while the other three patients showed extensive lesions with significant whole-brain atrophy. It may indicate that extensive leukodystrophy-like lesions with brain atrophy on MRI might be another risk factor of poor prognosis in adult MOG-E.

Our analysis demonstrated that MOGAD can present with chronically progressive course and leukodystrophy-like MRI pattern resembling genetic and neurodegenerative disorders, which may cause misdiagnosis and deferring of immune treatment. Therefore, we suggest to screen MOG-ab in patients with progressive cognitive or psychiatric symptoms and leukodystrophy-like MRI pattern to differentiate cMOG-E from untreatable diseases, which may facilitate treatment decision and prognosis. Besides, according to our experience and previous case reports, adult onset, chronically progressive encephalitic symptoms, delay of immunotherapy, and leukodystrophy-like MRI patterns with brain atrophy might be risk factors of poor prognosis in MOGAD patients.

In 310 AE patients we experienced, four patients with slow progression of encephalitic symptoms were identified. They showed different features with cMOG-E. In our study, MOG-E cohort had significantly higher proportion (30.8%) of slow progression course compared with AE (1.3%). In cAE cohort, patients tended to have abrupt symptoms (dystonia or seizures) after several months of slowly progressive cognitive impairment or psychiatric symptoms. In cMOG-E, abrupt symptoms were absent. With treatment of steroids, all cAE patients had remarkable improvement, while cMOG-E showed poor response. On MRI, three of four cAE patients showed normal results, but in cMOG-E, leukodystrophy-like lesions and brain atrophy were predominant phenotypes. In our study, cAE had better outcome than cMOG-E. This could be explained in the perspective of pathogenesis. Most of our cAE patients had positive LGI1-ab. They block the interaction of LGI1 with its receptors to disrupt protein-protein interaction, but they do not activate complement or cause damage to neurons. However, MOG-ab target the myelin sheath of axons and induce FcR-mediated antibody-dependent cellular cytotoxicity (33). As cognitive/psychiatric impairment mediated by different antibodies showed different prognosis, it is significant to differentiate them as early as possible. So, we suggest to test MOG-ab and AE-ab in patients with slowly progressive encephalitic symptoms.

Furthermore, we have two patients that showed tumefactive demyelinating lesion on MRI, which is defined as an acute large (>2 cm) tumor-like demyelinating lesion in the CNS that may occur with surrounding edema, mass effect, and ring enhancement (34). It was a not uncommon white matter change seen in brain MRI and was also reported in MOGAD cases in previous literature (35, 36). In this study, we incorporated tumefactive demyelinating lesion into MRI patterns of MOG-E, which enlarged brain MRI phenotypes of MOG-E on the basis of Hacohen’s study.

Co-existence of anti-NMDAR and anti-MOG antibodies also presented in the cohort (patients 4, 5, and 7). According to Fan’s study and Du’s study, 14.2% patients with anti-NMDAR encephalitis had overlapping MOGAD and 11.9% patients with MOGAD had overlapping anti-NMDAR encephalitis (37, 38). The exact pathogenesis of the co-occurrence is unclear (37–40), and it is difficult to tell which antibody is responsible for the encephalitis. Patient 5 presented with memory decline, seizures, and multifocal neurological symptoms. MRI showed multifocal poorly marginated lesions in cortical gray matter, subcortical white matter, and midline structures (**Figure 3G**). With positive MOG-IgG and NMDAR-IgG, we consider this case had MOGAD overlapping with anti-NMDAR encephalitis (1, 20). Patients 4 and 7 only presented with acute encephalitic symptoms without focal neurological sign. A cortical/subcortical lesion (**Figure 3H**) and slight atrophy of bilateral hippocampi (**Figure 3I**) were observed on MRI, respectively. With positive MOG-IgG and NMDAR-IgG, the two cases fulfilled the criteria for anti-NMDAR encephalitis (20), but not MOGAD for the absence of demyelination (1). However, in previous literature, cases who had MOG-IgG and encephalitis without demyelination had already been reported, and MOG-IgG was thought of as responsible for the isolated encephalitis (8). Moreover, for patients 4 and 7, the titer of MOG-IgG was constantly equal or higher than NMDAR-IgG during follow-ups, both in attack and in remission phases. It suggested that aside from NMDAR-IgG, MOG-IgG might also play a role in the pathogenesis of encephalitis. The coexistence of MOG-ab and NMDAR-ab may result from coexistence of MOG and NMDAR on oligodendrocytes. When autoantibodies attack one of them, the other one can be attacked simultaneously (17, 41, 42). Follow-up serological and imaging investigations may help to give the diagnostic propensity in the long run.

## Conclusions

The clinical and radiological presentations of MOGAD show a high degree of heterogeneity. With previous reports, our study further confirms that aside from acute and subacute onset, encephalitic symptoms associating with MOG-ab could have an insidious onset with slow progression. In radiology, lesion distribution can be various, involving cortex, hippocampus, corpus callosum, etc. MRI patterns of multifocal hazy/poorly marginated lesions and cortical encephalitis were common, but leukodystrophy-like white matter abnormalities with brain atrophy and tumefactive demyelinating lesions were also observed. Combined with this study and previous literature, we suggest that in patient with MOG-ab, adult onset, chronically progressive disease course, delay of immunotherapy, and leukodystrophy-like abnormalities with brain atrophy on MRI were possible risk factors of bad prognosis. In the differential diagnosis of rapidly progressive dementia, MOG-ab and AE-ab should be tested.

## Limitations

There are some areas that can be improved. Firstly, a larger cohort of patients can better represent the general features of MOG-antibody-associated encephalitis. Secondly, data collection through local evaluation combined with online follow-up methods needs to be spread, especially when we realize that it would not be convenient for disabled patients or to come to a tertiary hospital during coronavirus pandemic. That is the most important reason that some data, including follow-up MRI and MOG-IgG test, were absent.

## Data Availability Statement

The original contributions presented in the study are included in the article/**Supplementary Material**. Further inquiries can be directed to the corresponding author.

## Ethics Statement

The studies involving human participants were reviewed and approved by the ethics committee of the Xuanwu hospital, Capital Medical University. Written informed consent to participate in this study was provided by the participants’ legal guardian/next of kin.

## Author Contributions

JW drafted the work, collected data and designed the research project. ZQ and DL analyzed and interpreted data. XY analyzed data, and revised the grammar and sentences of the work. YD, LeG, AL, YS, CL, RG, LWu, LWa, LJ, DG, AZ, JJ, LH, MQ and LiG provided and interpreted patients’ data. HD interpreted data and drafted the work. JH interpreted data and provided critical advice. ZL designed and supervised the research project. All authors contributed to the article and approved the submitted version.

## Funding

This work was supported by the National Key R&D Program of China, Precision Medicine Program, Cohort study on nervous system diseases (grant number 2017YFC0907700), the National Science Foundation of China (grant number 81571633), and Beijing health system clinicians training plan (grant number 20143054).

## Conflict of Interest

The authors declare that the research was conducted in the absence of any commercial or financial relationships that could be construed as a potential conflict of interest.

## Publisher’s Note

All claims expressed in this article are solely those of the authors and do not necessarily represent those of their affiliated organizations, or those of the publisher, the editors and the reviewers. Any product that may be evaluated in this article, or claim that may be made by its manufacturer, is not guaranteed or endorsed by the publisher.
