# Supplementary material for: Clinical and Imaging Features of Patients With Encephalitic Symptoms and Myelin Oligodendrocyte Glycoprotein Antibodies
Source: Front Immunol. 2021 Oct 7;12:722404. doi: 10.3389/fimmu.2021.722404 (PMC8529193; doi:10.3389/fimmu.2021.722404)
Supplement: Supplementary file 1 [file DataSheet_1.pdf]

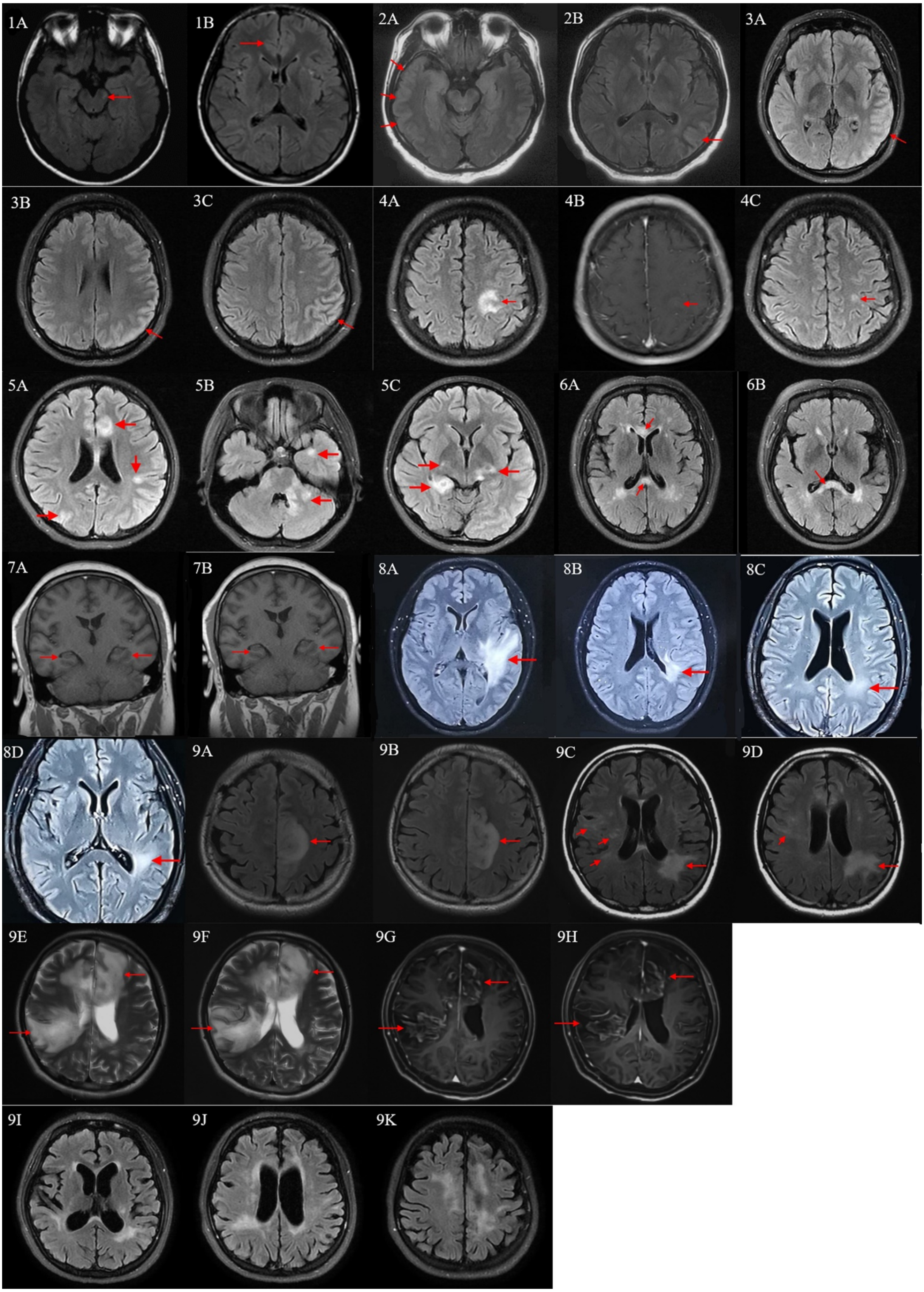
1A
1B
2A
2B
3A
3B
3C
4A
4B
4C
5A
5B
5C
6A
6B
7A
7B
8A
8B
8C
8D
9A
9B
9C
9D
9E
9F
9G
9H
9I
9J
9K

Supplementary figure 1: Brain MRI in patient 1~9.

Brain MRI of patient 1 showed lesions in brainstem (**1A**) and cingulate gyrus (**1B**). Brain MRI of patient 2 showed cortical and subcortical lesions in the right temporal (**2A**) and left frontal lobe (**2B**). Brain MRI of patient 3 showed cortical and subcortical lesions in the left frontal, temporal, parietal and occipital lobes (**3A, 3B** and **3C**). Brain MRI of patient 4 in 2020 showed cortical and subcortical lesions in the left frontal and parietal lobes (**4A**) with gadolinium enhancement (**4B**). After immune treatment, his MRI in 2021 showed prominent improvement (**4C**). Brain MRI of patient 5 showed multi-focal poorly marginated lesions in cortical gray matter, subcortical white matter and midline structures (**5A, 5B** and **5C**). Brain MRI of patient 6 showed lesions in corpus callosum (**6A** and **6B)**. Brain MRI of patient 7 in 2019 showed bilateral hippocampi atrophy (**7A**). After immunotherapy, no change was observed on his MRI in 2020 (**7B**). Brain MRI of patient 8 showed a tumefactive demyelinating lesion in the left temporal lobe (**8A** and **8B**). The lesion shrank after immunotherapy (**8C** and **8D**). Brain MRI of patient 9 during the first episode of encephalitis showed a tumefactive demyelinating lesion involving the left frontal and parietal lobe (**9A** and **9B**). Her MRI during the second episode of encephalitis showed multifocal hazy and poorly marginated lesions (**9C** and **9D**). During the third episode of encephalitis, the patient showed tumefactive demyelinating lesions on MRI involving the right temporal lobe and bilateral frontal lobes (**9E** and **9F**) with gadolinium enhancement (**9G** and **9H**). Her follow-up MRI showed white matter change and cortical brain atrophy (**9I**, **9J** and **9K**).

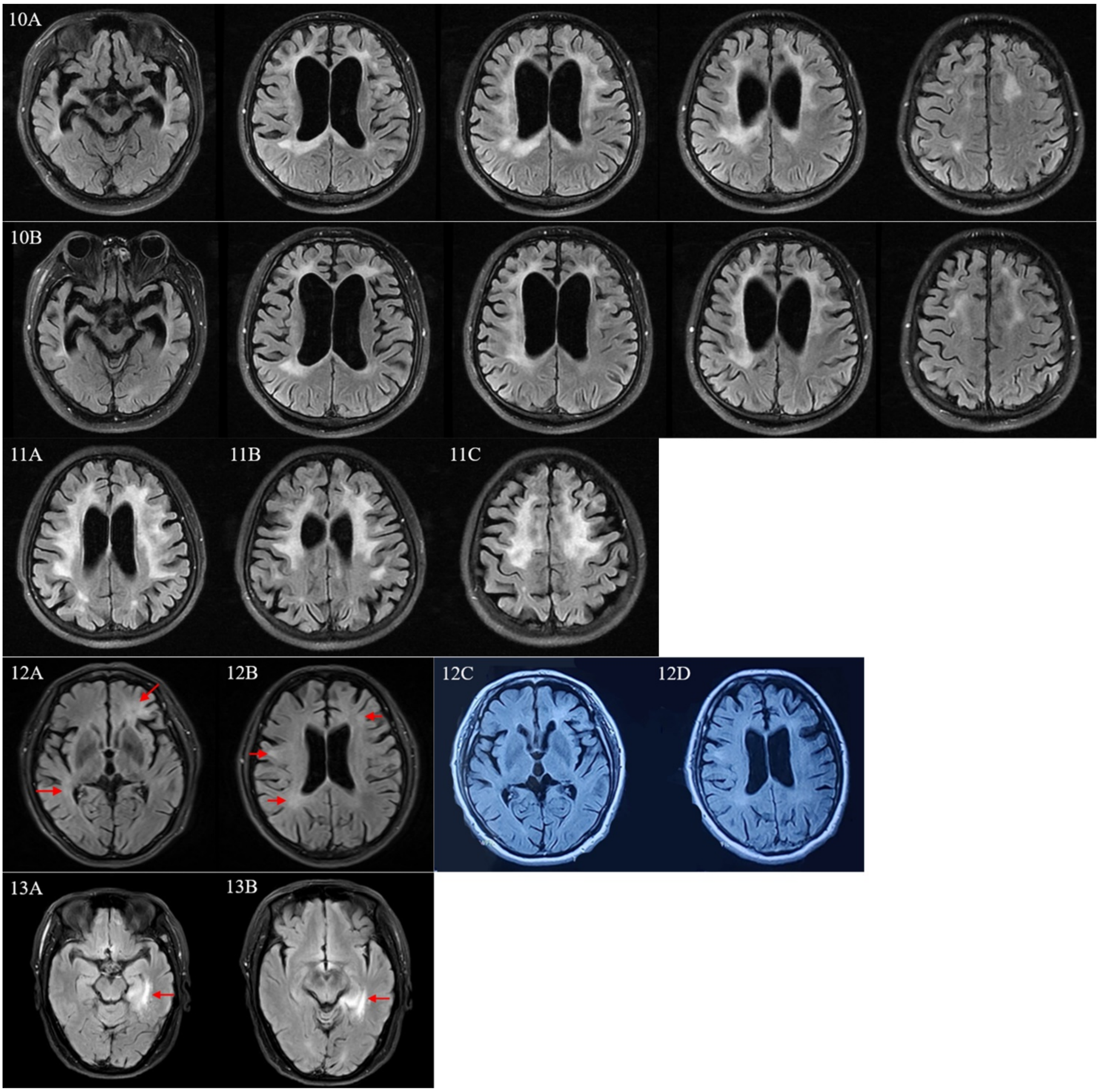

Supplementary figure 2: Brain MRI in patient 10~13.

Brain MRI of patient 10 in 2018 showed leukodystrophy-like white matter change with whole-brain atrophy (**10A**). After immunotherapy, the white matter change was improved on 2019 MRI (**10B**). Brain MRI of patient 11 showed leukodystrophy-like abnormalities with whole-brain atrophy (**11A**, **11B** and **11C**). Brain MRI of patient 12 in 2020 showed multifocal hazy and poorly marginated lesions with whole-brain atrophy (**12A** and **12B**). After immunotherapy, his MRI in 2021 did not show improvement of lesions (**12C** and **12D**). Brain MRI of patient 13 showed a lesion in the left hippocampus and parahippocampal gyrus (**13A** and **13B**).
