# Supplementary material for: Clinical and Imaging Features of Patients With Encephalitic Symptoms and Myelin Oligodendrocyte Glycoprotein Antibodies
Source: Front Immunol. 2021 Oct 7;12:722404. doi: 10.3389/fimmu.2021.722404 (PMC8529193; doi:10.3389/fimmu.2021.722404)
Supplement: Supplementary file 2 [file Table_1.pdf]

Supplementary table 1: Demographic, clinical characteristics and treatment of 13 patients with MOG-E.

Patient	Age (y)/ Sex	Disease duration at presentation (mo)	Episodes with encephalitic symptoms	Preceding infection	Form of onset, disease progression	Symptoms during encephalitic episodes					mRS/EDSS/ MMSE at nadir	Immunotherapy
						Decreased level of consciousness	Seizures	Cognitive impairment	Psychiatric symptoms	Other neurological symptoms		
1	13/M	89	E1	No	Acute, rapid	No	Yes	No	No	Headache	1/0/28	Steroids, IVIG
			E3	Yes	Acute, rapid	No	Yes	No	No	Headache, visual disturbance	1/0/29	IVIG, steroids, IFN beta-1b
2	46/M	3	E1	No	Acute, rapid	Yes, somnolence	Yes	No	Yes	Headache	2/2/28	Steroids, IVIG, MMF
3	24/F	0.2	E1	No	Acute, rapid	Yes, somnolence	Yes	Yes	Yes	Headache	2/2/25	IVIG
4	33/M	1	E1	No	Acute, rapid	No	Yes	No	No	None	1/0/29	IVIG, MMF
5	15/F	2	E1	Yes	Subacute, rapid	Yes, somnolence	Yes	Yes	No	Vertigo and limb weakness	3/2.5/24	IVIG, steroids
6	62/F	10	E1	No	Acute, slow	No	Yes	No	Yes	Limb weakness	4/8.5/21	IVIG, steroids
7	24/M	14	E1	Yes	Acute, rapid	No	No	Yes	Yes	None	2/2/25	Steroids, MMF
			E2	No	Acute, rapid	No	No	Yes	Yes	None	1/0/24	Steroids, MMF
8	39/M	9	E1	No	Subacute, rapid	No	No	Yes	No	Headache, limb weakness	3/3/15	Steroids, MMF
9	24/F	32	E1	No	Acute, rapid	No	Yes	No	No	Limb weakness, BON	4/7/26	Steroids
			E4	Yes	Acute, rapid	No	No	Yes	No	Limb weakness, LON	3/4/20	Steroids, MMF
			E6	No	Acute, rapid	No	No	Yes	Yes	None	3/3/18	Steroids, IVIG, RTX
**10**	30/M	24	E3	No	Insidious, slow	No	No	Yes	Yes	Bowel and bladder dysfunction	3/5.5/3	Steroids, MMF
**11**	55/F	24	E1	No	Insidious, slow	No	No	Yes	Yes	Bowel and bladder dysfunction	3/5/5	Steroids, MMF
**12**	52/M	24	E1	No	Insidious, slow	No	No	Yes	Yes	Headache, gait imbalance, bowel and bladder dysfunction	3/6/10	Steroids, MMF
**13**	59/F	12	E1	No	Insidious, slow	No	No	Yes	No	Headache	1/2/24	Steroids

BON=bilateral optic neuritis; E=episode; F=female; LON=left optic neuritis; M=male; mo=months; NA=not available; RON=right optic neuritis; w=weeks; y=years.

Supplementary table 2: Laboratory findings, MRI features and follow-up data of 13 patients with MOG-E.

Patient	Age (y)/ Sex	Episodes with encephalitic symptoms	CSF results			Serum/ CSF MOG-ab titer (1:)	Other positive antibodies	MRI features during encephalitic attacks (MRI pattern)	FU (mo)	mRS/EDSS /MMSE at last follow-up	Response to immunotherapy; outcome at last follow-up	Disease course
			WC (/uL)	Pro (mg/dL)	OB							
1	13/M	E1	160	52	NA	100/Neg	None	Lesions in cingulate gyrus (III)	53	0/0/28	Good response; full recovery	Highly relapsing
		E3	106	39	Pos		None	Lesions in cingulate gyrus and brainstem (III+I)				
2	46/M	E1	20	25	Neg	100/10	None	Cortical and subcortical lesions in the left frontal and right temporal lobe (III)	3	0/0/28	Good response; full recovery	Monophasic
3	24/F	E1	18	38	Neg	100/32	None	Cortical and subcortical lesions in the left frontal, temporal, parietal and occipital lobes (III)	3	0/0/29	Good response; full recovery	Monophasic
4	33/M	E1	9	34	Neg	100/100	NMDAR-ab 1:10 in CSF and serum	Cortical and subcortical lesions in the left frontal and parietal lobes (III)	10	0/0/29	Good response; full recovery	Monophasic
5	15/F	E1	11	39	Pos	100/10	NMDAR-ab 1:10 in CSF	Multi-focal poorly marginated lesions in cortical gray matter, subcortical white matter and midline structures (I+III)	33	0/0/28	Good response; full recovery	Monophasic
6	62/F	E1	1	36	Neg	100/1	Amphiphysin-ab, ANA and β2-GPI-ab	Lesions in corpus callosum (I)	24	0/0/26	Good response; full recovery	Monophasic
7	24/M	E1	3	47	Neg	100/1	NMDAR-ab 1:100 in CSF, RF	Atrophy in bilateral hippocampi (III)	25	0/0/29	Good response; full recovery	Relapsing
		E2	1	34	Neg		NMDAR-ab 1:10 in CSF	Atrophy in bilateral hippocampi (III)				
8	39/M	E1	180	53	Neg	100/10	None	A tumefactive demyelinating lesion in the left temporal lobe (IV)	45	1/2/29	Good response; full recovery from encephalitic symptoms, partial recovery from ON	Relapsing
9	24/F	E1	76	37	Pos	100/1	None	A tumefactive demyelinating lesion involving the left frontal and parietal lobe (IV)	10	3/4/23	Partial response; remarkable improvement from cognitive and psychiatric symptoms, mild recovery from visual impairment	Highly relapsing
		E4	NA	NA	NA		None	Multifocal hazy and poorly marginated lesions involving the left temporal, parietal and occipital lobes; small non-specific lesions in the right hemisphere and corpus callosum (I)				

		E6	21	29	Neg		None	Tumefactive demyelinating lesions involving the right temporal lobe and bilateral frontal lobes (IV)				
**10**	30/M	E3	5	35	Neg	100/Neg	None	Leukodystrophy-like pattern with whole-brain atrophy (II+III)	34	3/5.5/3	Mild response; worse cognitive and psychiatric abnormalities, slight improvement on MRI lesions	From relapsing to progressive
**11**	55/F	E1	1	51.2	Neg	100/10	None	Leukodystrophy-like pattern with whole-brain atrophy (II+III)	4	3/5/5	Mild response; mild improvement on psychiatric symptoms	Progressive
**12**	52/M	E1	31	62	Neg	320/Neg	None	Mild white matter change with mild whole-brain atrophy (I+III)	14	3/6/10	No response; worse mood lability, cognitive impairment and gait imbalance, no improvement on MRI lesions	Progressive
**13**	59/F	E1	2	23	Neg	100/Neg	None	A lesion in the left hippocampus and parahippocampal gyrus (III)	24	1/2/24	Mild response; no further deterioration in cognitive impairment	Monophasic

β2-GPI=β2-glycoprotein I; ab=antibody; ANA=anti-nuclear antibody; CSF=cerebrospinal fluid; DEX=dexamethasone; EDSS=Expanded Disability Status Scale; F=female; FU=follow-up; IFN=interferon; IV=intravenous; IVIG=intravenous immunoglobulin; IVMP=intravenous methylprednisolone; M=male; MMF=mycophenolate mofetil; mo=months; MOG=myelin oligodendrocyte glycoprotein; MP=methylprednisolone; mRS=modified Rankin scale; NA=not available; Neg=negative; NMDAR=N-methyl-D-aspartate receptor; OB=oligoclonal bands; ON=optic neuritis; Pos=positive; Pro=protein; PSL=prednisolone; RF=rheumatoid factor; RTX=rituximab; WC=white cell count; y=years.
